# Stratification of ovarian tumor pathology by expression of programmed cell death-1 (PD-1) and PD-ligand- 1 (PD-L1) in ovarian cancer

**DOI:** 10.1186/s13048-018-0414-z

**Published:** 2018-05-30

**Authors:** Maureen L. Drakes, Swati Mehrotra, Monica Aldulescu, Ronald K. Potkul, Yueying Liu, Anne Grisoli, Cara Joyce, Timothy E. O’Brien, M. Sharon Stack, Patrick J. Stiff

**Affiliations:** 1 Cardinal Bernardin Cancer Center, Oncology Research Institute, Department of Medicine, Loyola University Chicago, Bldg. 112, Room 232, 2160 South First Avenue, Maywood, IL 60153 USA; 2 Department of Pathology, Stritch School of Medicine, Loyola University Chicago, Maywood, IL USA; 3 Department of Obstetrics and Gynecology, Loyola University Chicago, Maywood, IL USA; 4 Department of Chemistry & Biochemistry, Harper Cancer Research Institute, University of Notre Dame, South Bend, IN USA; 5 Department of Public Health Sciences, Loyola University Chicago, Maywood, IL USA; 6 Department of Mathematics & Statistics, and Institute of Environmental Sustainability, Loyola University Chicago, Chicago, IL USA

**Keywords:** Programmed cell death-1, Programmed cell death-1 ligand, High grade disease, Cancer immunotherapy, Ovarian cancer

## Abstract

**Background:**

Ovarian cancer is the major cause of death among gynecologic cancers with 75% of patients diagnosed with advanced disease, and only 20% of these patients having a survival duration of five years. Treatments blocking immune checkpoint molecules, programmed cell death (PD-1) or its ligand PD-ligand- I (PD-L1) have produced a beneficial and prolonged effect in a subgroup of these patients. However, there is debate in the literature concerning the prognostic value of the expression of these molecules in tumors, with immunotherapy responsiveness, and survival.

We evaluated the immune landscape of the ovarian tumor microenvironment of patients, by measuring the impact of the expression of tumor PD-1, PD-L1 and infiltrating lymphocytes on stage and grade of tumors and survival, in a cohort of 55 patients with gynecologic malignancies. Most patients under study were diagnosed with advanced disease ovarian cancer.

**Results:**

Our studies revealed that a low density of PD-1 and of PD-L1 expressing cells in tumor tissue were significantly associated with advanced disease (*P* = 0.028 and *P* = 0.033, respectively). Moreover, PD-L1 was expressed significantly more often in high grade tumors (41.5%) than in low grade tumors of patients (7.7%) (*P* = 0.040). The presence of CD3 or of FoxP3 infiltrating cells with PD-L1 in patient tumors did not impact the significance of the association of PD-L1 with high grade tumors (P = 0.040), and our analyses did not show an association between the presence of PD-1 or PD-L1 and survival.

**Conclusions:**

We conclude that a subgroup of advanced disease ovarian cancer patients with high grade tumors, expressing PD-L1, may be prime candidates for immunotherapy targeting PD-1 signaling.

**Electronic supplementary material:**

The online version of this article (10.1186/s13048-018-0414-z) contains supplementary material, which is available to authorized users.

## Background

The early signs of ovarian cancer are asymptomatic and thus approximately 75 % of cases are detected in the advanced metastatic stages. Conventional management strategies for advanced disease include cytoreductive surgery and chemotherapy. Most current treatments are not curative for patients with advanced disease and hence survival for this category of patients is low [1]. It is estimated that in 2017 there will be 22,440 new cases of ovarian cancer in the Unites States, and that 14,080 patients will die due to this disease [2]. Approximately 80 % of patients diagnosed with late stage ovarian cancer die within five years.

To provide more effective treatment options for patients, several clinical trials are ongoing using novel single and combination regimens to improve survival. For cancer therapy, there have been several distinct landmarks in the development of new therapies and FDA approved treatments over the last decade [3]. However, even with the current treatment options a considerable number of patients are not yet receiving adequate therapy for the management of advanced stage ovarian cancer and other malignancies.

The development and optimization of the use of novel therapies such as immunotherapy, requires an in-depth understanding of specific target molecules and cellular interactions in tumors. Early efforts in immunotherapy can be traced to 1891, in which administration of intra-tumoral injections of bacteria led to a shrinkage of patients’ tumor [4, 5]. Since then, significant progress has been made in the field [6]. One of the recent highlights in novel treatment options for cancer has been the targeting of immune checkpoint inhibitory molecules [7–9]. Immune checkpoints are critically important in health and disease. They represent co-signaling pathways which are either costimulatory or coinhibitory. In the body, linkage of coinhibitory receptor and ligand suppresses T cell receptor signaling, and limits immune responses. Whereas this function of checkpoint inhibitory molecules is beneficial during resolution of infection, or in the development of self tolerance to prevent autoimmune conditions [10–12], ligation of checkpoint inhibitory molecules can be a powerful and unwanted mechanism of immunosuppression in cancer [13–15]. Since the successful introduction and FDA approved use of an antibody targeting checkpoint inhibitory molecule cytotoxic T lymphocyte associated-4 (CTLA-4) (Ipilimumab; Yervoy®) in patients with unresectable or metastatic melanoma in 2011 [16], this agent is now in use in over 40 countries. Attention has more recently focused on another checkpoint inhibitory molecule programmed cell death-1 (PD-1) and its ligand programmed cell death-1 ligand (PD-L1) [17–19].

Antibodies inhibiting PD-1 and PD-L1 have recently been FDA approved for the treatment of cancer. For example, the agent nivolumab (Opdivo®) is approved for unresectable or metastatic melanoma, non-small cell lung cancer (NSCLC), Hodgkin’s lymphoma and renal cell carcinoma. Pembrolizumab (Keytruda®) is FDA approved for melanoma and NSCLC, and a blocking anti-PD-L1 antibody Atezolizumab (Tecentriq®) is also FDA approved for unresectable bladder cancer and for NSCLC. Blockade of this pathway is particularly useful in patients as it is applicable to a wide range of cancers, and because it induces anti-tumor immune responses capable of targeting mutated proteins [20]. Importantly, treatment targeting PD-1 signaling has fewer high grade toxicities than other immunotherapies [13, 21].

Medical centers are currently utilizing these agents in ongoing clinical trials for various cancers, including ovarian cancer [7, 22, 23]. Initial reports of some trials show promising objective response rates (ORR) for the treatment of ovarian cancer with anti-PD-1 antibody nivolumab (ORR of 15%, *n* = 20 patients), and pembrolizumab (ORR 11.5%, *n* = 49), or an anti-PD-L1 antibody avelumab (ORR 10%, *n* = 124) [3, 24]. Those responding often had durable responses, suggesting that if we could identify the subgroup that might typically respond, we could advance the therapeutic options in this subgroup of ovarian cancer patients.

PD-1 is primarily expressed on CD4+ and CD8+ T cells and is associated with T cell exhaustion [11, 12, 14]. PD-L1 is expressed on many cell types including tumor cells and macrophages, including those with an immunosuppressive phenotype [12, 25, 26]. Ligation of PD-L1 on tumor cells with PD-1 on T cells, for example, abrogates T cell proliferation, diminishes T cell activation and leads to a predominance of a T helper 2 (Th2) cytokine tumor microenvironment, with a pro-tumor propensity. Antibody blocking of PD-1 or PD-L1 restores T cell proliferative and cytotoxic functions, and induces a T-helper 1 (Th1) phenotype, thereby re-invigorating T cells, with resulting potent anti-tumor capacity [14, 27, 28].

The immune mechanisms of disease improvement with administration of checkpoint inhibitory molecules are not well understood. Clinically, there is also ongoing debate over which patients will benefit from this therapy, whether patients who respond initially will continue to show complete responses (CR) or partial responses (PR), and whether patients’ tumors need to express PD-1 and/ or PD-L1 in abundance, to predict beneficial responses to checkpoint inhibitory molecule blocking therapy targeting these molecules. At the present time, there are more questions than answers.

As a study of the immune microenvironment of ovarian cancer patient tumors offers insight into the baseline immune landscape associated with patient survival and tumor pathology, and implicates broader scope for targeting these molecules in combination studies with conventional therapy and with other novel therapies, we undertook these present investigations. We primarily selected advanced disease ovarian cancer patients for study, since this group typically have poor outcome with standard therapy, and our future goal in translational medicine is to address the need for novel alternative treatment options in this patient sector. We evaluated the expression and localization of PD-1 and PD-L1 in a cohort of ovarian cancer formalin fixed paraffin embedded (FFPE) tumor sections, and investigated whether the relative expression levels of these molecules can be relevant patient prognostic indicators. We also studied the impact of tumor infiltrating lymphocytes (TILS) along with these checkpoint molecules, on patient status including tumor grade, disease stage and survival post diagnosis.

## Methods

### Patients

Patients underwent surgery between 2003 and 2006 at Loyola University Medical Center (LUMC) for ovarian and other gynecologic associated cancers. Tissues were embedded in paraffin blocks for patient diagnosis to characterize stage and grade of cancer in tissue sections, and blocks were stored in the Department of Pathology, LUMC. After receiving approval by the Institutional Review Board (IRB) for the Protection of Human Subjects, we selected a cohort of 55 patients for study, most of whom were diagnosed with advanced disease ovarian cancer (Table 1). Patient histories in the LUMC medical records were evaluated by two investigators and data collected for parameters including: age, date of birth, date of diagnosis, pre-treatment status before surgery, cancer stage, tumor grade, date of last encounter, and whether the patient was alive or deceased. Dates of death were retrieved from the patients’ medical records when this date was available, or found by a search on a website such as http://www.dobsearch.com/death-records/.

**Table 1:** Categories of patient tumors

Stage^a^	Tumor type	Histological subtype	No. of patients
Low (I/ II)	All Ovarian	papillary mucinous cystadenocarcinoma mixed adenocarcinoma, serous and endometroid papillary serous adenocarcinoma papillary serous carcinoma serous carcinoma	1 1 2 4 1
High (III/ IV)	Ovarian Ovarian Ovarian Fallopian tube Peritoneum Peritoneum Endometrium Endometrium Omentum	poorly differentiated serous carcinoma papillary serous carcinoma papillary serous adenocarcinoma papillary serous carcinoma papillary serous carcinoma papillary serous adenocarcinoma papillary serous carcinoma serous carcinoma papillary serous carcinoma	1 24 10 1 4 1 1 1 2
Total			55

^a^Tumor stage reflects the International Federation of Gynecology and Obstetrics (FIGO) classification

### Antigen revealing

Formalin fixed paraffin embedded (FFPE) tissue sections (4 μm) were adhered to glass slides using tissue from a single patient on each slide for detection of PD-L1, PD-1, CD3 and CD8 by immunohistochemistry (IHC). For staining of FoxP3 on T cells, patient tissue arrays were constructed from the paraffin embedded blocks and adhered on a total of 2 glass slides with a core of tissue from each of 27 or 28 patients, as well as control tissues. Positive control thymus tissue highly expressed the molecules/ markers under study. Negative control tissue was sections of benign ovarian disease such as polycystic ovarian disease. Sections on slides were de-paraffinized in xylene and then rehydrated in a series of decreasing concentrations of alcohols. Antigen retrieval for PD-L1 and PD-1 was performed by boiling slides in a pressure cooker for 5 min in Universal HIER retrieval agent (ab 208,572, Abcam, Cambridge, MA) at a 1X concentration. Sections were washed in 0.1% tween in Dulbecco’s phosphate buffered saline (DPBS; 1X, Lonza, Walkersville, MD) and then blocked in 0.4% hydrogen peroxide in DPBS, followed by blocking in 10% goat serum (S1000, Vector Laboratories, Burlingame, CA) for 1 h.

Antigen retrieval for FoxP3, CD3 and CD8 was performed by boiling sections in a pressure cooker for 5 min in Reveal Decloaker (RV1000G1, Biocare Medical, Concord, CA). After washing in DPBS, sections were blocked in 0.4% hydrogen peroxide in DPBS for 20 min, 10% goat serum or 10% horse serum (S1000 or S2000 respectively, Vector Laboratories) for 20 min, and then in Avidin/ Biotin blocking reagents (SP 2001, Vector Laboratories) to further reduce non-specific staining of primary antibody (FoxP3, CD3, or CD8).

### Identification and assessment of antigens in patient sections

Tissue sections were incubated overnight in 5% blocking serum with or without primary antibody at a pre-determined and optimized dilution. PD-1 (ab137132, Abcam) and PD-L1 (ab205921, Abcam) were used at 1:500 dilution for IHC staining. The next day sections were washed in 0.1% tween in DPBS, and an amplifier polymer detection system specific for rabbit anti-human primary antibodies (ab 20,901, Rabbit specific IHC polymer detection kit; HRP/ DAB) added according to the manufacturers’ guidance.Tissue was also stained overnight with primary antibodies for FoxP3 (236A/E7; ab 20,034, 1:1600 dilution, Abcam), CD8 (C8/144B; 1:100 dilution, Cell Marque, Rocklin, CA 1:1000 dilution) and CD3 (F7.2.38; 1:1000 dilution, Dako, Glostrup, Denmark). Sections were washed in DPBS and a biotinylated secondary antibody for peroxidase (PK 6102, Vector Laboratories) added for 30 min, followed by an avidin-biotin peroxidase complex and enzyme reagent (ABC, Vector laboratories). All sections were washed in DPBS and developed in Vector NovaRED (SK4800) or diaminobenzidine (DAB; SK4100, Vector Laboratories). Sections were counterstained in hematoxylin and rehydrated in xylene, followed by alcohol, then mounted in Vectamount H-5000 (Vector Laboratories).

Tumor sections were examined by pathologists SM and MA to investigate the frequency of occurrence of markers, the degree of staining intensity and location of tumor cells or lymphocytes expressing each molecule. A customized scoring system was developed by the abovementioned pathologists to obtain a numerical score to represent the average frequency of antigens as visualized over 7–10 high power fields (hpf) of IHC stained tissue sections *(*Table 2), where “0” was the lack of expression, and “4” represented the highest frequency of expression of molecules in sections. In addition to the scoring pattern shown in Table 2, in statistical analysis, combined PD-1 was assigned as a mathematical score which was derived by adding the observed pathology scores (0–2) for T-PD-1 and S-PD-1 in each patient section.

**Table 2:** Pathological interpretation of IHC stained tissue

Marker	Scoring criteria based on cells/ high power fields^a^
T-PD-1	0 = < 1; 1 = 1–10, 2= > 10–50 and 3= > 50
S-PD-1	0 = < 1, 1 = 1–25; 2= > 25–50 and 3= > 50
PD-L1	0 = < 1, 1= > 1–5, 2= > 5–10 and 3= > 10
CD3	0 = < 5, 1 = 5–15, 2= > 15–25, 3= > 25–40 and 4= > 40
CD8	0 = < 1, 1 = 1–25, 2= > 25–50 and 3= > 50
FoxP3	0 = < 1, 1 = 1–5, 2= > 5–15, 3= > 15–25 and 4= > 25 cells/ hpf

^a^Based on the known frequency of each marker in various cancer tissues, each stained section was observed over 7–10 hpf and given an average numerical value to represent the scoring frequency based on the categorization in Table 2. PD-1 was assigned 2 different scores to reflect the observed localization of this molecule in tumor tissue. The percentage of PD-1 stained TILs completely enclosed by tumor epithelial cells was termed tumor-PD-1 (T-PD-1). PD-1 stained lymphocytes in the stromal compartment were classified as stromal-PD-1 (S-PD-1)

In some statistical analysis PD-1 and PD-L1 expression was classified as low (score of 1) or high frequency (score of 2–4) to decipher correlations between the levels of expression of these molecules and parameters studied.

### Statistical analysis

Patient O/S was displayed visually in Kaplan Meier plots and significance of differences by strata were determined with Log Rank tests. The frequency of occurrence of each marker was graded on a scale from 0 to 4 (Table 2), and Cochran Armitage tests used to determine the statistical significance of trends by patient characteristics including age, cancer stage and tumor grade. Associations between the presence of PD-1, PD-L1, CD3, CD8 and FoxP3 positive cells with patient age at the time of diagnosis, cancer stage, or tumor grade were determined with chi-square or Fisher’s exact tests as appropriate. Hazard ratios for overall survival (O/S) were determined from univariable Cox proportional hazard regression models for each patient characteristic and each marker. Analyses were performed using SAS 9.4 (SAS Institute, Cary, NC).

## Results

### Patient characteristics

The cohort consisted of 55 patients as follows: ovarian (45 patients), fallopian tube (1 patient), peritoneum (5 patients), endometrium (2 patients) and omentum (2 patients) cancer tissue blocks. Patients were diagnosed as stage I or stage II (stage 1 or 2, low/ early stage; 9 patients) and stage III or IV (stage 3 or 4, high stage or advanced; 46 patients) disease (Table 1). One patient did not have known cancer grade, and of the remaining, about three quarters of patients had tumor grade 3 (*n* = 41, 75.9%).The mean age of patients at the time of diagnosis was 61 years (standard deviation = 12), with a range of 26 to 85 years. At the time of last follow up, 6 patients were alive, 9 patients were lost to follow up (3 of whom were deceased at an unknown date), and 40 other patients had recorded dates of death, 38 of whom had died as a cause of ovarian cancer. The median length of survival time for all patients was 3.10 years (95% confidence interval (CI): 2.24–5.19).

### Localization of checkpoint inhibitory molecules in ovarian cancer tissue

**Fig. 1:**
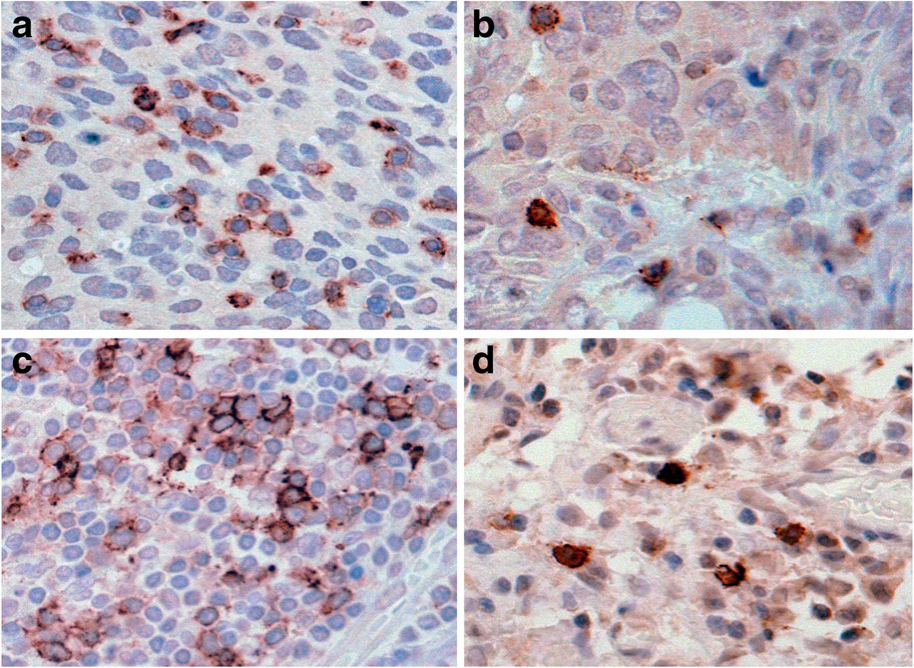
Distinct IHC staining patterns of PD-1 positive cells in the stroma and epithelium of tissue. In ovarian cancer tissue sections, tumor infiltrating lymphocytes (TILS) exhibiting strong membranous and cytoplasmic staining for PD-1 are apparent as clusters (**a**) or single scattered cells (**b**). TILS are also seen in aggregates (**c**) and as single cells (**d**) in the reactive stroma associated with tumor

We studied the distribution of PD-1 and PD-L1 in ovarian cancer tissue sections by IHC staining. Sections were visualized and staining evaluated by methods as outlined in Table 2. Results showed that PD-1 in ovarian cancer tissue was localized primarily to cell membranes. PD-1 stained cells appeared to be primarily tumor infiltrating lymphocytes (TILs), with varying degrees of intensity of staining and frequency of occurrence of these cells. Additionally, this staining was strikingly evident in two separate compartments of the tumor microenvironment, thus we scored this stain in two different categories. Intraepithelial TILs completely enclosed by tumor epithelial cells and positive for PD-1 were designated as tumor PD-1 cells (T-PD-1). These cells were either clustered (Fig. 1a), or scattered in a less dense pattern in the tumor epithelium (Fig. 1b). Cells which stained positive for PD-1 in the stromal compartment were termed stromal PD-1 (S-PD-1). In the stroma, there were aggregates of cells staining positive for PD-1 (Fig. 1c), or regions of fewer cells staining for this molecule (Fig. 1d). Table 3 shows a summary of scores for each PD-1 classification as well as the frequency of PD-1 observations in tissue in the cohort of 55 patients studied. A total of 48 (87%) patients expressed PD-1 on cells, whilst 40 (73%) patients expressed PD-1 in both the epithelial (T-PD-1) and stromal (S-PD-1) compartments (data not shown).

**Table 3:** Occurrence of checkpoint inhibitory molecules and TILS in tumors

Molecules in tumor sections^a^	Frequency					Total patients positive
	0	1	2	3	4	
T-PD-1	10	35	10	0	0	45
S-PD-1	12	39	4	0	0	43
PD-L1	37	14	3	1	0	18
CD3	2	23	18	5	7	53
CD8	17	15	18	5	0	38
FoxP3	7	12	15	19	2	48

^a^Tumor sections of 55 ovarian and related cancers were IHC stained for molecules as indicated. Sections were visualized by microscopy and a pathological score assigned from 0 to 4, based on the frequency of expression of each molecule, where “0” indicates the absence and “4” the highest frequency of expression

**Fig. 2:**
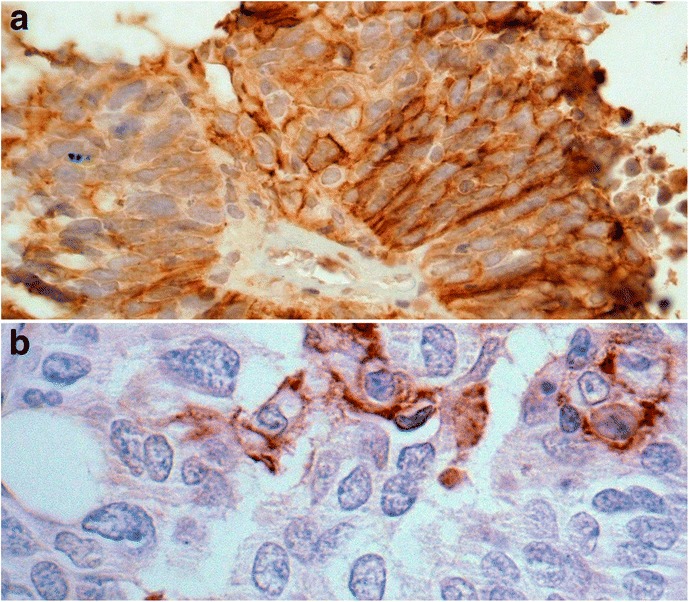
Membranous staining of PD-L1 positive cells in ovarian tumors. Diffuse membranous staining of almost all ovarian cancer cells (**a**) versus focal strong membranous staining in a few scattered tumor cells positive for PDL1 (**b**)

In tissue sections, PD-L1 was localized to the cell membrane. In lesions of some patients there were solid tumor aggregates with diffuse membranous staining of these cells for this marker (Fig. 2a). PD-L1 was also identified in scattered tumor cells with focal strong membranous staining (Fig. 2b). Additionally, in some sections PD-L1 staining was observed in cells with the morphology of immune cells (not shown). Staining for this marker was positive in approximately one-third (18 of 55, 33%) of patients with ovarian cancer.

### Variable expression of tumor infiltrating lymphocytes in ovarian cancer

**Fig. 3:**
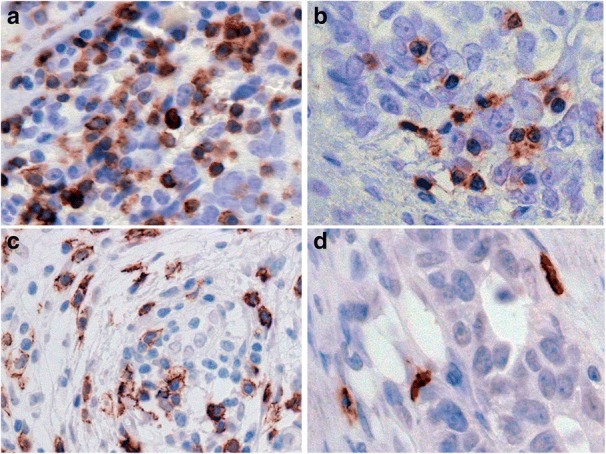
High and low density of TILs in ovarian tumors. IHC staining of T cell subsets in patients’ FFPE tissue sections. CD3 exhibiting diffuse strong staining in clusters of tumor infiltrating lymphocytes (**a**) versus focal staining in scattered TILs in less dense areas (**b**). Distribution of CD8 T cells in clusters and as single cells is apparent in **c** and **d** respectively

**Fig. 4:**
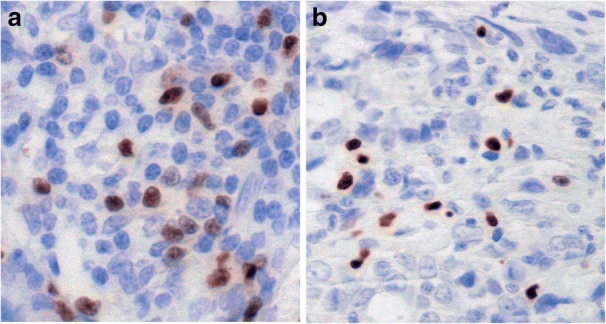
Distribution of FoxP3 expressing T regulatory cells in ovarian tumors. Intense nuclear staining of FoxP3 positive lymphocytes in a focal arrangement (**a**). Other cases (**b**) showed nuclear staining of fewer cells positive for this marker

Immunocompetent TILS and FoxP3 T regulatory cells, are strategically located in ovarian cancer tissue. We sought to determine whether the levels of CD3, CD8 and/ or FoxP3 T cells in the tumors of patients would alter the potential role of checkpoint inhibitory molecules as predictors of disease pathology or of outcome. In FFPE tissue sections, of all T cells, CD3 positive T cells were found in highest frequency, as expected (Table 3), sometimes staining intensely. Some patients had an abundance of these cells, while other cases showed a scattered arrangement (Fig. 3a and b respectively). Staining for CD8 T cells was widely observed in patient sections as membranous reactivity, either with a dense distribution, or with lower frequency (Fig. 3c and d respectively). FoxP3 staining of cells was revealed as strong nuclear reactivity, either in clustered foci in some patient sections (Fig. 4a), or scattered in the tumor stroma in other cases (Fig. 4b). FoxP3 positive cells were present in 48 of 55 patients.

### Clinical parameters as predictors of patient prognosis

We examined trends between age of patients at the time of diagnosis, tumor grade or tumor stage with overall survival. Significant parameters for these associations are represented in Kaplan Meier plots (Additional file 1: Figure S1). We found that patients diagnosed with ovarian cancer when over 60 years of age had a higher hazard of death (HR: 2.63, 95% CI: 1.34–5.16) and were significantly more likely to die as a cause of disease than patients diagnosed at a younger age (*P* = 0.005), (Additional file 2: Table S1). Additionally, patients diagnosed with advanced disease (stage III or IV) had a higher hazard of death (HR: 3.70, 95% CI: 1.28–10.76) and were significantly more likely to die than patients diagnosed in the early stages of the disease (*P* = 0.016). Tumor grade had no significant association with patient survival (Additional file 2: Table S1).

Similar analysis was performed to determine survival estimates as a function of the expression of each marker. It was found that while survival was similar in the first few years of follow-up for those with or without PD-L1, those with PD-L1 present in tissue sections appeared to have a survival advantage with increased time after diagnosis, even though this trend was not statistically significant (Additional file 3: Figure S2A). The presence of PD-1 and of CD3 showed modest but insignificant trends toward improved survival (Additional file 3**:** Figure S2 B and C). CD8 or FoxP3 did not show a significant association with survival, even though for high expression of FoxP3 there was a trend towards decreased survival (data not shown).

### The presence of PD-L1 is positively associated with high grade tumors

We undertook detailed analysis to determine how the presence of checkpoint inhibitor molecules, PD-1 or PD-L1 correlated with patient history. We did not find any significant associations between the presence of PD-1, with patient age at the time of diagnosis, stage of cancer or tumor grade (Table 4).

**Table 4:** Patient history and presence of PD-1 and PD-L1

		combined PD-1^a^		PD-L1	
	No. Patients	n (%)	*p*-value	n (%)	*P*-value
Age at diagnosis					
< 60	27	24 (88.9)	0.99	9 (33.3)	0.93
≥ 60	28	24 (85.7)		9 (32.1)	
Stage of cancer					
I-II	9	9 (100.0)	0.58	5 (55.6)	0.13
III-IV	46	39 (84.8)		13 (28.3)	
Tumor grade					
1–2	13	12 (92.3)	0.99	1 (7.7)	*0.040*
3	41	35 (85.4)		17 (41.5)	

^a^The presence of PD-1 in the tumor epithelial or stromal compartment (combined PD-1), and PD-L1 was defined as the occurrence of these molecules (scores 1, 2, 3, or 4) in tissue sections. Tumor grade was unknown for one patient. Significant *P*-values are indicated in italics

Only one patient with low tumor grade expressed PD-L1, thus 17/18 (94%) patients who expressed PD-L1 in their ovarian tumors had a tumor grade of 3. Thus PD-L1 was expressed significantly more often in patients with high grade tumor than in those with low grade tumor (*n* = 54; 41.5% versus 7.7%, *P* = 0.040) (Table 4). We did not observe any other significant associations with the presence of any other molecules studied as single predictors, with age at the time of diagnosis, stage of disease or tumor pathology.

Further analysis of data showed that the presence of CD3 or of FoxP3 infiltrating cells together with PD-L1 in patient tumors did not impact the significance of the association of PD-L1 with high grade tumors (*P* = 0.040) (Table 5**).** There was borderline significant association between the presence of S-PD-1 cells together with FoxP3 positive cells in tumors with high grade (*P* = 0.075). The presence of TILs and of cells expressing PD-1 or PD-L1 occurring together in ovarian tumors had no impact on disease stage (Table 5), or on survival (data not shown).

**Table 5:** Association with immune markers, grade and stage

	No. Patients	n (%) with stage III-IV	*P*-value	n (%) with grade 3	*P*-value
Overall	55	46 (83.6)		41/54 (75.9)	
^a^Presence with CD3					
T-PD-1 + CD3	44	35 (79.5)	0.18	31 (72.1)	0.26
S-PD-1 + CD3	43	34 (79.1)	0.18	34 (81.0)	0.13
Combined PD-1 + CD3	47	38 (80.9)	0.33	34 (73.9)	0.66
PD-L1 + CD3	18	13 (72.2)	0.13	17 (94.4)	*0.040*
Presence with FoxP3					
T-PD-1 + FoxP3	41	33 (80.5)	0.42	30 (75.0)	0.99
S-PD-1 + FoxP3	41	33 (80.5)	0.42	33 (82.5)	0.075
Combined PD-1 + FoxP3	44	36 (81.8)	0.67	33 (76.7)	0.99
PD-L1 + FoxP3	18	13 (72.2)	0.13	17 (94.4)	*0.040*

^a^Evaluation of the association of the presence of PD-1 or PD-L1 together with CD3 and FoxP3 T cells on advanced disease and on high grade tumors. Significant *P*-values are indicated in italics

### Low frequency of expression of PD-1 and of PD-L1 correlates with advanced ovarian cancer

We further investigated whether high or low frequency of expression of PD-1 correlated with the stage or grade of cancer. Based on our scoring pattern for the occurrence of PD-1 in the tumor epithelium (T-PD-1) or in the stroma (S-PD-1), we initially analyzed observations in these compartments separately. As expected, the frequency of PD-1 expressing cells in tumor tissue was in general not as high as those expressing CD3 or CD8 (Table 3). A higher percentage of patients with early stage cancer were more likely to have a higher frequency of expression (pathology score of 2) of T-PD-1 than in patients with advanced disease, but this difference was not significant (*P* = 0.13, data not shown). In the case of S-PD-1, a higher percentage of patients with early stage cancer had higher levels (frequency score of 2, 22%) of S-PD-1 than for advanced disease patients (frequency score of 2, 2%) (*P* = 0.033) (Additional file 4: Table S2).

A significantly higher percentage (33%) of early stage patients had a higher frequency (score 3 and 4) of combined PD-1 (PD-1 in the tumor epithelium and/or stroma) than those with advanced disease (17%) (*P* = 0.028, Table 6). Thus, a high density of PD-1 was significantly associated with early stage disease diagnosis, and a low PD-1 density was associated with advanced disease.

**Table 6:** Patient tumor and combined frequency of PD-1 expression

	No. Patients	0	1	2	3	4	*P*-value
		^a^Combined PD-1 Level, n (%)					
Stage of cancer							
I-II	9	0 (0.0)	0 (0.0)	6 (66.7)	1 (11.1)	2 (22.2)	*0.028*
III-IV	46	7 (15.2)	8 (17.4)	23 (50.0)	7 (15.2)	1 (2.2)	
Tumor grade							
1–2	13	1 (7.7)	4 (30.8)	7 (53.8)	0 (0.0)	1 (7.7)	0.54
3	41	6 (14.6)	4 (9.8)	21 (51.2)	8 (19.5)	2 (4.9)	

^a^Combined PD-1 was derived by adding the observed pathology scores for tumor PD-1 (T-PD-1) and S-PD-1 in each patient tumor section. Significant *P*-value in table is indicated in italics

PD-L1 was expressed in 33% of the patient cohort. Scoring for PD-L1 was performed by scoring regions of intense or moderate staining for PD-L1 in either stromal or tumor epithelial compartment, and an average of cells stained over 7 hpfs taken into account. Most patients (5/9; 56%) with early stage disease expressed PD-L1. A lower percentage of patients with advanced disease expressed PD-L1 (13/46; 28%) (Table 7). Most patients (11/13; 85%) with advanced disease who expressed PD-L1, expressed this molecule in tumors with a score of 1 (on a scale of 0–3). A higher percentage of patients with early stage disease (22%) expressed high levels of PD-L1 (frequency score 2 or 3) in comparison with patients with advanced disease (4.3%) (Table 7). There were no significant trends in frequencies of CD3, CD8 or FoxP3 with age at the time of diagnosis, stage of disease, or tumor grade (data not shown).

**Table 7:** Patient tumor characteristics and frequency of PD-L1 expression

	No. Patients	0	1	2	3	*P*-value
		PD-L1 Level, n (%)				
Stage of cancer						
I-II	9	4 (44.4)	3 (33.3)	1 (11.1)	1 (11.1)	*0.033*
III-IV	46	33 (71.7)	11 (23.9)	2 (4.3)	0 (0.0)	
Tumor grade						
1–2	13	12 (92.3)	1 (7.7)	0 (0.0)	0 (0.0)	*0.033*
3	41	24 (58.5)	13 (31.7)	3 (7.3)	1 (2.4)	

Significant *P*- values in table are indicated in italics

## Discussion

Ovarian cancer is usually diagnosed in the advanced metastatic stages. Treatment of advanced stage disease with conventional therapies is only sufficiently effective in a limited number of patients, thus in about 80 % of these patients there is disease progression or disease recurrence and death, within five years of diagnosis. In many cancers, investigators are focusing on the development of novel therapies as alternative and more robust options to existing therapies. Whereas conventional therapies primarily focus on the destruction of tumor cells, many novel therapies are designed to stimulate immune cells to elaborate augmented anti-tumor immune responses. In this respect, checkpoint immune inhibitory molecules have come full circle over the last decade for cancer immunotherapy.

In a normal functioning immune system, T cell activating and inhibitory receptors balance immune tolerance, and the amplification of immune responses. In the body, immune checkpoints are designed to reduce autoimmune responses, or to attenuate immune responses which were elaborated after infections [10–12, 14, 27, 28]. In cancer, blocking of immune checkpoint molecules with antibodies is a novel and promising therapy, as it potentiates anti-tumor immune responses in patients [9, 13, 15, 19].

The first of these checkpoint inhibitory molecules to be targeted for blocking in therapy, and is now FDA approved for cancer therapy, is CTLA-4 [16, 17]. Therapy design is based on the following principle. Binding of costimulatory molecules CD80 or CD86 on antigen presenting cells to CD28 on T cells delivers a positive costimulatory signal contributing to T cell activation. On the contrary, linkage of CD80 or CD86 to CTLA-4 (a molecule closely related to CD28) results in inhibition of immune responses, and exhausted T cells, which are less able to proliferate or to secrete T helper 1 (Th1) cytokines [29] . Blocking of this inhibitory pathway with anti-CTLA-4 antibodies, results in re-invigorated T cells with greater proliferative function, and durable anti-tumor potency [30, 31]. By similar rationale, there are several other immune checkpoints in the immune system, which are now being targeted in cancer clinical trials. In the case of blocking PD-1 signaling, there are currently about nine different antibodies in cancer clinical trials targeting the PD-1/ PD-L1 pathway [3, 17].

This study was designed to better understand the ovarian cancer tumor microenvironment (TME) with relation to the localization and frequency of PD-1, PD-L1 and TILS in the tumors of ovarian or related cancer patients, diagnosed with advanced disease. Firstly, results showed that patients’ age was an independent prognostic factor in survival, with patients over 60 years of age more likely to die than those diagnosed when younger than 60 years. This may be due to the fact that younger patients can tolerate more aggressive surgery and chemotherapy than older patients. Additionally, as expected, disease stage was also an independent prognostic factor in outcome, such that patients diagnosed with advanced disease had a lower median survival than patients diagnosed with early stage disease. These findings are in agreement with those of other investigators [32].

To shed light on the relevance of PD-1 and PD-L1 in ovarian cancer outcome, we studied the abundance of these molecules in the TME. PD-1 was compartmentalized in the stroma and in the tumor epithelium, and this molecule was expressed in 87% of tumors. PD-L1 was only present in the tumors of 33% of patients. Patients who expressed PD-L1 had a trend towards survival, as did those expressing PD-1 or CD3, even though these trends were not significant. In our cohort we did not find a significant association with FoxP3 and survival. The presence of PD-L1 and FoxP3 together in high grade tumors showed the same level of association as the presence of PD-L1 alone. Some studies have reported that FoxP3 positive cells in ovarian tumors is negatively associated with outcome, however a meta-analysis of 7 ovarian cancer studies with a total of 869 patients, did not find FoxP3 TILS in ovarian cancer to be a significant prognostic indicator [33].

In cancer there are conflicting reports concerning the expression patterns of PD-1 in patients’ tumors and the association with survival, with either positive or a negative association [34–36]. One recent report found that PD-1 positive TILS and /or PD-L1 positive tumor cells had a positive association with survival of ovarian cancer patients [37].

The expression of PD-L1 in tumors was shown to be positively associated with survival in NSCLC [38] and in ovarian cancer [37]. On the contrary, others report a negative prognostic impact of PD-L1 expression in ovarian cancer [39, 40] and breast cancer [41]. In a review and meta-analysis of 17 studies using data of 2869 head and neck cancer (HNC) patients, authors found that there was no significant association between the expression of PD-L1 on survival in HNC patients [42]. Additionally, similar analysis of reports with NSCLC patients also did not show a significant association with PD-L1 expression and survival [43]. Taken together, this indicates that the prognostic impact of PD-1/ PD-L1 expression in tumors is not yet established. Here, our studies did not find a significant association between survival and PD-1 or PD-L1 expression in ovarian cancer.

Differences in reports of the expression of these molecules and associations with survival may be attributed to several reasons. Firstly, it is possible that there may be different survival outcomes due to the site of cancer. For example, Paulsen and colleagues [38] found that whereas in a cohort of patients a high density of PD-1 and PD-L1 had a favorable impact on NSCLC, this association was not present when these molecules were studied at metastatic sites such as lymph nodes of the same patients. This is highly likely because each cancer site has a different immune landscape, and levels of soluble molecules such as IFN-γ which is a strong regulator of PD-L1 expression [44, 45].

Secondly, differences in reports may be due to variations in staining protocols across individual laboratories. Many investigators report difficulty in IHC staining for PD-L1. In these present investigations, we initially used tumor arrays to study the expression of PD-L1 and PD-1 in tumors. When optimizing our staining protocol, we found that it was difficult to select cores which had a good representation of tumor and stromal areas for accurate visualization of the density of these molecules in tissue arrays. Therefore, in this study we used whole tumor sections for the identification of these molecules, as is done for patient diagnosis. For molecules such as PD-L1 especially, which is not widely expressed in tumor tissues, reports in which IHC staining was performed using tumor cores may give different findings than reports from other laboratories in which staining was performed using whole tissue sections.

Thirdly, the use of different primary antibody clones to identify PD-1 or PD-L1 in tumor tissue in each laboratory, may also lead to variability in staining of sections and in interpretation. Finally, manual staining protocols in comparison with automated staining may further contribute to variations in staining interpretation.

Finally, additional parameters which may alter the expression of these molecules in tumors, is the administration of treatments such as chemotherapy to patients prior to surgery. However, at our center, for patients diagnosed with ovarian and related gynecologic cancer, the primary treatment is most often surgery for removal of tumors. Patients then undergo courses of standard therapy such as chemotherapy. Consistent with this practice, we found that a study of our patient cohort treatment plans revealed that only 3 of 55 (5.5%) patients had chemotherapy in the interval before surgery.

A low frequency of PD-1 in tumors was associated with advanced disease. This association between low PD-1 density and advanced disease was only significant when measuring S-PD-1 or combined PD-1, whereas low T-PD-1 density alone was not associated with advanced disease. Although beyond the scope of this study, this finding raises the possibility that T-PD-1 and S-PD-1 positive cells may perform unique immunosuppressive roles in the ovarian TME.

PD-L1 expression was almost exclusively restricted to high grade tumors, such that there was a positive and significant association between PD-L1 and high grade tumors. This finding may be of translational significance in selecting patients for therapy blocking PD-1/ PD-L1 signaling, and we suggest that patients with high grade tumors, with pre-existing PD-L1 expression might be excellent candidates for therapy blocking this pathway. In support of this idea, a recent report shows that in an ongoing study of urothelial bladder cancer patients, treatment with durvalumab (MED14736; an anti-PD-L1 antibody) resulted in improved outcome in PD-L1 tumor positive patients. In pre-treatment tumor biopsies patients, 40 patients were PD-L1 positive and 21 patients negative for PD-L1. In 42 evaluable patients, the ORR was 31.0% (95% CI, 17.6 to 47.1), the ORR was 46.4% (95%CI, 27.5 to 66.1) in the PD-L1 positive patient subgroup, and 0% (95% CI, 0.0 to 23.2) in patients negative for PD-L1 [46].

We suggest that due to the conflicting reports concerning the impact of PD-1 and PD-L1 on survival in cancer patients, a future larger study is needed investigating these molecules in ovarian tissue, with standardized protocols and defined cut off points for positive staining and scoring criteria across centers, to minimize study variations. Even so, the potency of patient responses to PD-1/ PD-L1 blocking antibody therapy may be influenced by the density of other pre-existing or emerging checkpoint molecules in tumors, including T-cell immunoglobulin and mucin-domain containing-3 (TIM-3), lymphocyte-activation gene 3 (LAG-3) and V-domain Ig suppressor of T cell activation (VISTA). Other parameters such as the presence of myeloid derived suppressor cells, levels of Th2 cytokines (for example IL-10) and of indoleamine 2,3-dioxygenase (IDO) can also limit anti-cancer immune responses to therapy blocking PD-1/ PD-L1 [19, 47, 48]. Furthermore, genetic alterations within the tumor (including DNA rearrangements, mutations, deletions and insertions) alter tumor mutational loads, and it is reported that tumors with high mutational loads have the greatest response to checkpoint inhibitory blockade therapy [17, 49, 50].

Finally, due to the multiplicity of factors regulating ORR, we believe that antibody therapy targeting the PD-1/ PD-L1 pathway in ovarian cancer will be of maximum efficacy when used in combination with other treatment regimens. Such treatments include standard therapy, immunotherapy blocking other checkpoint inhibitory molecules, dendritic cell vaccines, chimeric antigen receptor (CAR) T cell therapy, or targeted therapy, all of which can downregulate other immune suppressive mechanisms in patients, concomitantly.

## Conclusions

Treatments inhibiting PD-1 and PD-L1 are beneficial only in some ovarian cancer patients. Our studies reveal that a low density of PD-1 and of PD-L1 expressing cells in tumor tissue are significantly associated with advanced disease, and that PD-L1 is expressed significantly more in high grade tumors than in low grade tumors. We conclude that a subgroup of advanced disease ovarian cancer patients with high grade tumors, bearing PD-L1, may be the best candidates for immunotherapy targeting PD-1 and/ or PD-L1.

## Additional files

Additional file 1: **Figure S1.** Survival estimates by patient age and tumor grade. Kaplan Meier survival analysis to estimate overall survival in patients as a function of age (A) or tumor stage (B). Patient survival was displayed visually in Kaplan Meier plots and significance of differences determined with Log Rank tests. (PPTX 427 kb)

Additional file 2: **Table S1.** Univariable hazard ratios for Cox proportional hazards models (DOCX 25 kb)

Additional file 3: **Figure S2.** Survival estimates by expression of immune molecules. Kaplan Meier survival analysis to estimate overall survival in patients expressing PD-L1 (A), PD-1 (B) and CD3 (C) in tumor sections. (PPTX 456 kb)

Additional file 4: **Table S2.** Patient tumor and frequency of S-PD-1 expression (DOCX 25 kb)

## Abbreviations

CR: complete response
CTLA-4: cytotoxic T lymphocyte associated-4
FFPE: formalin fixed paraffin embedded
HNC: head and neck cancer
hpf: high power fields
IHC: immunohistochemistry
NSCLC: non-small cell lung cancer
O/S: overall survival
ORR: objective response rate
PD-1: programmed cell death-1
PD-L1: programmed cell death-1 ligand
PR: partial response
S-PD-1: stromal PD-1
Th1: T helper-1
TILS: tumor infiltrating lymphocytes
TME: tumor microenvironment
T-PD-1: Tumor PD-1
