# Supplementary material for: Stratification of ovarian tumor pathology by expression of programmed cell death-1 (PD-1) and PD-ligand- 1 (PD-L1) in ovarian cancer
Source: J Ovarian Res. 2018 May 30;11:43. doi: 10.1186/s13048-018-0414-z (PMC5975524; doi:10.1186/s13048-018-0414-z)
Supplement: Supplementary file 1 — Figure S1. Survival estimates by patient age and tumor grade. Kaplan Meier survival analysis to estimate overall survival in patients as a function of age (A) or tumor stage (B). Patient survival was displayed visually in Kaplan Meier plots and significance of differences determined with Log Rank tests. (PPTX 427 kb) [file 13048_2018_414_MOESM1_ESM.pptx]

## Slide 1
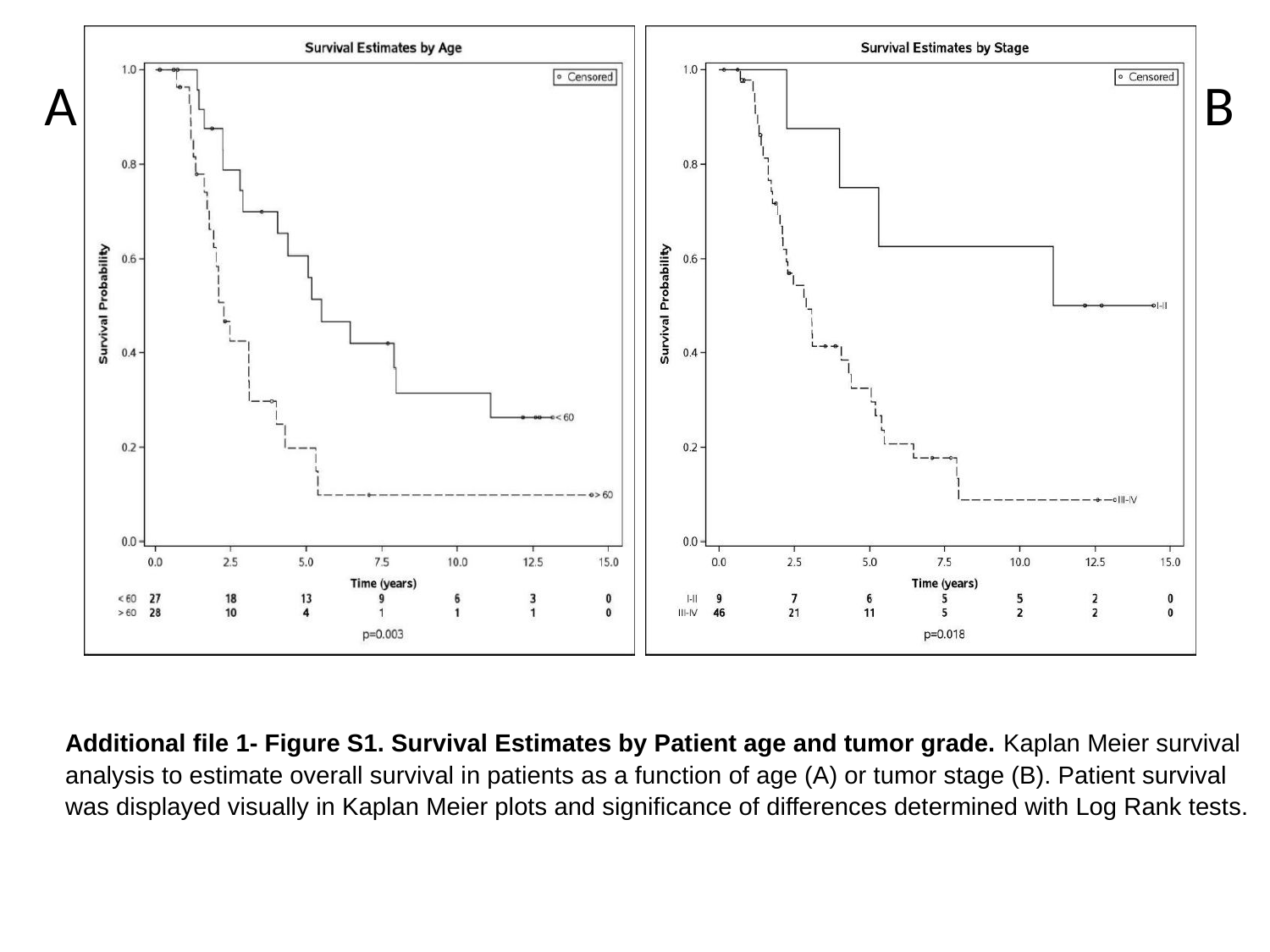

A
B
Additional file 1- Figure S1. Survival Estimates by Patient age and tumor grade. Kaplan Meier survival analysis to estimate overall survival in patients as a function of age (A) or tumor stage (B). Patient survival was displayed visually in Kaplan Meier plots and significance of differences determined with Log Rank tests.
1
