# Supplementary material for: Stratification of ovarian tumor pathology by expression of programmed cell death-1 (PD-1) and PD-ligand- 1 (PD-L1) in ovarian cancer
Source: J Ovarian Res. 2018 May 30;11:43. doi: 10.1186/s13048-018-0414-z (PMC5975524; doi:10.1186/s13048-018-0414-z)
Supplement: Supplementary file 2 — Table S1. Univariable hazard ratios for Cox proportional hazards models (DOCX 25 kb) [file 13048_2018_414_MOESM2_ESM.docx]

**Additional file 2. Table S1: Univariable hazard ratios for Cox proportional hazards models**

|  | ^a^Hazard Ratio (95% CI) | *P*-value |
| --- | --- | --- |
| Age at diagnosis |  | 0.005 |
| < 60 | 1 (Reference) |  |
| ≥ 60 | 2.63 (1.34, 5.16) |  |
| Stage of cancer |  | 0.016 |
| I-II | 1 (Reference) |  |
| III-IV | 3.70 (1.28, 10.76) |  |
| Tumor grade |  | 0.43 |
| 1-2 | 1 (Reference) |  |
| 3 | 1.39 (0.61, 3.18) |  |
| Presence of molecules^b^ |  |  |
| S-PD-1 | 1.26 (0.55, 2.88) | 0.58 |
| T-PD-1 | 1.07 (0.49, 2.35) | 0.87 |
| PD-1 | 1.33 (0.52, 3.43) | 0.55 |
| PD-L1 | 0.64 (0.30, 1.33) | 0.23 |
| FoxP3 | 1.25 (0.44, 3.53) | 0.67 |
| CD3 | 0.87 (0.12, 6.40) | 0.89 |
| CD8 | 1.21 (0.61, 2.40) | 0.59 |

^a^Cox proportional hazard models were fitted to determine clinical or immune
parameters which may impact survival.
^b^reference category is absence of molecules
confidence interval; CI.
