# Supplementary material for: Stratification of ovarian tumor pathology by expression of programmed cell death-1 (PD-1) and PD-ligand- 1 (PD-L1) in ovarian cancer
Source: J Ovarian Res. 2018 May 30;11:43. doi: 10.1186/s13048-018-0414-z (PMC5975524; doi:10.1186/s13048-018-0414-z)
Supplement: Supplementary file 3 — Figure S2. Survival estimates by expression of immune molecules. Kaplan Meier survival analysis to estimate overall survival in patients expressing PD-L1 (A), PD-1 (B) and CD3 (C) in tumor sections. (PPTX 456 kb) [file 13048_2018_414_MOESM3_ESM.pptx]

## Slide 1
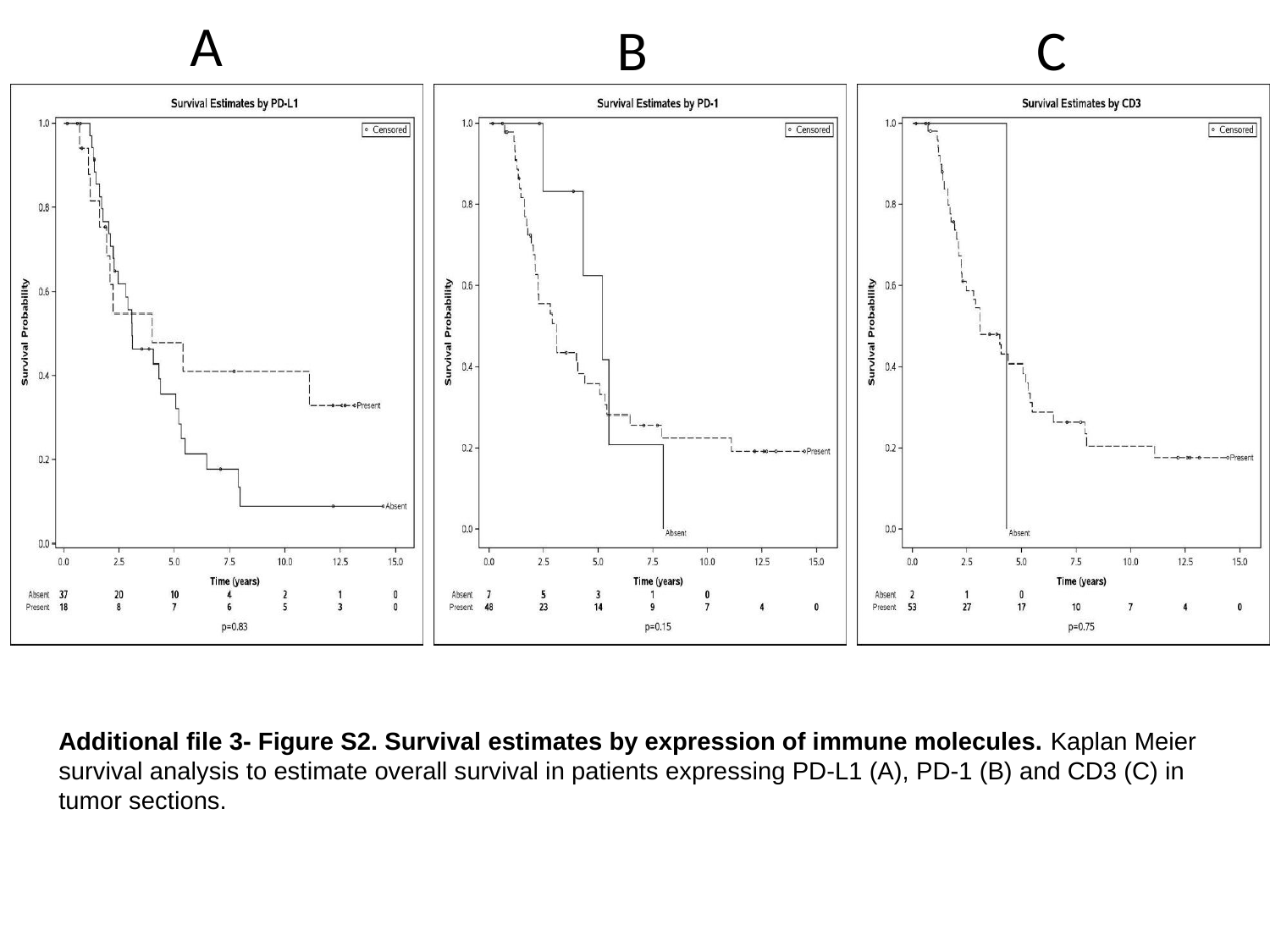

A
B
C
Additional file 3- Figure S2. Survival estimates by expression of immune molecules. Kaplan Meier survival analysis to estimate overall survival in patients expressing PD-L1 (A), PD-1 (B) and CD3 (C) in tumor sections.
1
