# Supplementary material for: Stratification of ovarian tumor pathology by expression of programmed cell death-1 (PD-1) and PD-ligand- 1 (PD-L1) in ovarian cancer
Source: J Ovarian Res. 2018 May 30;11:43. doi: 10.1186/s13048-018-0414-z (PMC5975524; doi:10.1186/s13048-018-0414-z)
Supplement: Supplementary file 4 — Table S2. Patient tumor and frequency of S-PD-1 expression (DOCX 25 kb) [file 13048_2018_414_MOESM4_ESM.docx]

**Additional file 4. Table S2: Patient tumor and frequency of S-PD-1 expression**

|  | **No. Patients** | **0** | **1** | **2** | ***P*-value** |
| --- | --- | --- | --- | --- | --- |
|  |  | **S-PD-1, n (%)** | | |  |
| Stage of cancer |  |  |  |  |  |
| I-II | 9 | 0 (0.0) | 7 (77.8) | 2 (22.2) | **0.033** |
| III-IV | 46 | 12 (26.1) | 32 (69.6) | 2 (4.3) |  |
| Tumor grade |  |  |  |  |  |
| 1-2 | 13 | 5 (38.5) | 7 (53.8) | 1 (7.7) | 0.24 |
| 3 | 41 | 7 (17.1) | 31 (75.6) | 3 (7.3) |  |
